# Single-cell RNA sequencing in orthopedic research

**DOI:** 10.1038/s41413-023-00245-0

**Published:** 2023-02-24

**Authors:** Tao Wang, Ling Wang, Liping Zhang, Yubin Long, Yingze Zhang, Zhiyong Hou

**Affiliations:** 1 Department of Orthopedic Surgery, Third Hospital of Hebei Medical University, Shijiazhuang, Hebei PR China; 2 Orthopedic Research Institute of Hebei Province, Third Hospital of Hebei Medical University, Shijiazhuang, Hebei PR China; 3 Department of Orthopedic Oncology, Third Hospital of Hebei Medical University, Shijiazhuang, Hebei PR China; 4 Department of Physiology, Hebei Medical University, Shijiazhuang, Hebei PR China; 5 NHC Key Laboratory of Intelligent Orthopedic Equipment (Third Hospital of Hebei Medical University), Hebei, PR China

**Keywords:** Pathogenesis, Multihormonal system disorders

## Abstract

Although previous RNA sequencing methods have been widely used in orthopedic research and have provided ideas for therapeutic strategies, the specific mechanisms of some orthopedic disorders, including osteoarthritis, lumbar disc herniation, rheumatoid arthritis, fractures, tendon injuries, spinal cord injury, heterotopic ossification, and osteosarcoma, require further elucidation. The emergence of the single-cell RNA sequencing (scRNA-seq) technique has introduced a new era of research on these topics, as this method provides information regarding cellular heterogeneity, new cell subtypes, functions of novel subclusters, potential molecular mechanisms, cell-fate transitions, and cell‒cell interactions that are involved in the development of orthopedic diseases. Here, we summarize the cell subpopulations, genes, and underlying mechanisms involved in the development of orthopedic diseases identified by scRNA-seq, improving our understanding of the pathology of these diseases and providing new insights into therapeutic approaches.

## Introduction

Osteoarthritis (OA), lumbar disc herniation (LDH), rheumatoid arthritis (RA), fractures, tendon injuries, spinal cord injury (SCI), heterotopic ossification (HO), and osteosarcoma (OS) are common bone-related disorders that affect tens of thousands of people.^1–8^ Due to their high treatment costs, these diseases impose substantial burdens on individuals and societies.^1–3,5–8^ An increasing number of studies have investigated the molecular mechanisms of these disorders and effective treatments for patients suffering from them. Orthopedic disorders are caused by a series of complicated pathological processes, but the cellular origins and pathogenesis mechanisms of these diseases have not yet been fully elucidated. The development of specific and effective treatment options beyond surgery for these orthopedic disorders is urgently needed.

**Fig. 1:**
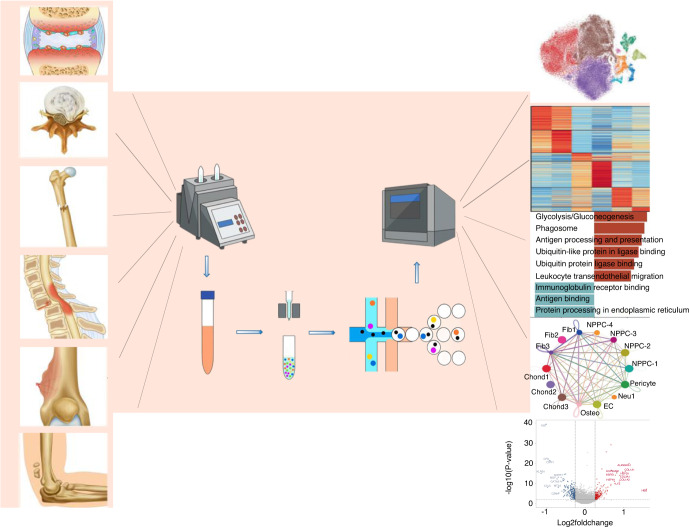
The brief workflow of single-cell RNA sequencing for these orthopedic disorders

Bulk RNA sequencing (RNA-seq) has been used as a powerful tool for investigating these orthopedic disorders^9^ because it can provide an overview of biological components, enable identification of new genes, and elucidate related signaling networks involved in disease processes. However, this method does not reveal the identity and function of specific cellular subsets or the differences in gene expression at the single-cell level,^10^ which limits our understanding of the biology and pathology of these diseases. Single-cell RNA sequencing (scRNA-seq) allows researchers to investigate transcriptomic cell-to-cell variation, identify different cell types, and obtain new perspectives on pathological processes, which contributes to a comprehensive understanding of the pathophysiology of diseases and facilitates the development of effective therapeutic regimens.^11,12^ The brief workflow of scRNA-seq is as follows (Fig. 1): (1) single-cell preparation; (2) isolation of single cells and library preparation; (3) sequencing and primary analysis; and (4) data visualization and interpretation. Critical to the advancement of scRNA-seq technology is the ongoing optimization of solutions to two key problems: (1) the isolation and capture of single cells and (2) the reverse transcription and amplification of cDNA with limited mRNA.^13^

ScRNA-seq has four major advantages over bulk RNA-seq in biological research. First, this approach can reveal cellular heterogeneity or specific subclusters; second, it can depict the trajectories of different cell states, transitions, and differentiation in tissue during physiological and pathological processes; third, scRNA-seq can be used to construct heterogeneous cellular signaling models according to enrichment analyses; and fourth, it can reveal the regulatory networks among clusters.^14^ ScRNA-seq findings have improved our overall understanding of these disorders and have thus provided promising treatment regimens to prevent, interfere with, or even cure these diseases. Numerous reviews have reported on the applications of scRNA-seq in other contexts, such as in tumor,^15^ kidney,^16^ heart,^17^ and brain^18^ research.

However, few studies have reviewed the applications of scRNA-seq in orthopedic research. Therefore, we searched orthopedic-related studies using the following keywords: “single-cell RNA sequencing,” “osteoarthritis,” “lumbar disc herniation,” “rheumatoid arthritis,” “fractures,” “tendon injury,” “spinal cord injury,” “heterotopic ossification,” or “osteosarcoma” in the English PubMed, Embase, and Cochrane Library databases up to November 2022. We introduce these orthopedic diseases according to their prevalence (Table 1). It is well known that OA, LDH, and RA, the three most prevalent orthopedic diseases, are degenerative, chronic, and painful disorders that impact numerous individuals. Fractures and tendon injuries are common orthopedic traumas that may lead to disabilities and numerous complications, such as nonunion or rerupture. SCI, which has a lower incidence than fractures or tendon injuries, is a life-threatening orthopedic injury that can lead to paralysis and even death. HO is a relatively rare complication that limits the motion of extremities after fractures. OS, the least common of these orthopedic disorders, is a highly aggressive and malignant bone tumor with a high relapse rate and a low survival rate that seriously affects teenagers, presenting a substantial health resource burden for families and societies. In this paper, we reviewed these orthopedic disorders based on scRNA-seq findings regarding new markers, new subtypes, function of novel cell types, potential mechanisms, cell-fate transitions, and cell‒cell interactions (Table 1).

**Table 1:** Characteristics of orthopedic diseases according to disease prevalence

Diseases	Prevalence	Main Location	New markers	New subtypes	Function of the novel cell type	Potential Mechanisms	Cell-fate transition	Cell-cell interactions
OA	6.8% to 28%^1^	Keen, hip	-	√	√	√	-	-
LDH	1% to 5%^2^	Cervical, lumbar	√	√	√	√	-	√
RA	0.5% to 1%^3^	Hand, wrist, keen	√	√	√	√	-	-
fracture	3.21 of 1 000 population^4^	bones	-	√	√	√	√	-
tendon injury	about 1 of 1 000 people^5^	hand, achilles tendon	-	√	√	√	√	√
SCI	1.3‰ of general population^6^	Spinal cord	-	√	√	√	-	-
HO	20% of the patients with forearm fractures^7^	fracture section	-	√	√	√	√	-
OS	5.6 cases per million in children under the age of 15^8^	metaphysis of long bones	-	√	√	√	-	√

*LDH* lumbar disc herniation, *OA* osteoarthritis, *RA* rheumatoid arthritis, *OS* osteosarcoma, *SCI* spinal cord injury, *HO* heterotopic ossification

### Applications of scRNA-seq in osteoarthritis

OA is a common chronic and degenerative disease resulting from the inflammation and degradation of articular cartilage,^19,20^ which is derived from condensed mesenchymal stem cells (MSCs) and composed of chondrocytes.^21^ Synovial joints are complex anatomical structures that include articular cartilage, synovium, fibrous capsules, and ligaments.^22^ Previous studies have identified four chondrocyte subclusters (proliferative chondrocytes, prehypertrophic chondrocytes, hypertrophic chondrocytes, and fibrocartilage chondrocytes)^23^ and cartilage progenitor cells^24^ in OA patients. The use of scRNA-seq to reveal cellular heterogeneity and new subtypes represents a significant step toward understanding the pathogenic mechanisms of OA.

Ji et al.^25^ discovered three novel subpopulations: effector chondrocytes, regulatory chondrocytes, and homeostatic chondrocytes. Effector chondrocytes were related to the tricarboxylic acid cycle and amino acid metabolism, indicating that they provided cellular energy. Regulatory chondrocytes were involved in many signaling pathways, suggesting that they might play crucial roles in regulating OA progression. Homeostatic chondrocytes exhibited high expression of genes related to cell cycle regulation, metabolic processes, and development, implying that these cells might contribute to the circadian clock in the context of OA progression (Table 2). These three populations exerted a protective effect on OA progression because protective genes were highly expressed. Furthermore, two subclusters of hypertrophic chondrocytes were also identified by Ji et al.^25^: hypertrophic chondrocyte-A with high expression of genes related to cartilage development and connective tissue development and the hypertrophic chondrocyte-B subset with high expression of genes related to extracellular matrix (ECM) organization, ossification, and mineralization. These findings have improved our understanding of the effect of hypertrophic chondrocytes on the development of OA (Table 2). Chou et al.^26^ identified two undescribed populations of chondrocytes: reparative chondrocytes and prefibrochondrocytes (Table 2). The former type exhibited a high reparative ability due to the high expression of genes related to ECM signaling and collagen fibril organization, whereas the latter type might play an essential role in ERK signaling due to the high expression of fibroblast-related genes and *IL11*. Sebastian et al.^27^ first reported nine chondrocyte subtypes (Ucma^high^, Cytl1^high^, Chil1^high^, Mef2c^high^, Krt16^high^, Tnfaip6^high^, S100a4^high^, Neat1^high^, and divC chondrocyte clusters) in articular cartilage from the healthy mouse knee joint. Several genes with regulatory functions were expressed in the Cytl1^high^, Chil1^high^, Tnfaip6^high^, and S100a4^high^ clusters, while genes related to protein synthesis and mRNA metabolism were highly expressed in the Ucma^high^ and Krt16^high^ clusters. Additionally, Sebastian et al.^27^ investigated the similarities between mouse and human chondrocyte transcriptomes and discovered that the Cytl1^high^, Chil1^high^, Krt16^high^, S100a4^high^, and divC clusters in mice were similar to human effector chondrocytes, regulatory chondrocytes, proliferative chondrocytes, fibrocartilage chondrocytes, and cartilage progenitor cell clusters, respectively. Sebastian et al.^27^ found early molecular changes in OA chondrocytes versus normal chondrocytes, including upregulation of *Mmp3*, *Mmp13*, *Ptgs2*, *Inhba*, *Sfn*, and *Il11* and downregulation of *Cytl1*, *Errfifi1*, and *Il17b*. Lv^28^ was the first to identify a ferroptotic chondrocyte cluster in human OA samples and noted that activating TRPV1 could prevent chondrocytes from undergoing ferroptosis by partially upregulating *GPX4*. These findings may have new implications for the development of effective OA drugs and delivery systems.

**Table 2:** Summary of major subclusters

Author	Subclusters	Evidence level	Disease	Resource	Comparative groups	Functions	Verification
***Chondrocytes***							
***RegCS***							
Ji et al.^25^	RegCS	III	OA	Human	Different OA stages	regulating OA progression	IHC
Chou et al.^26^	RegCS	III	OA	Human	non-OA vs OA	Negative regulation of biological process; Cellular response to metal ion	immunofuorescence staining; RT-qPCR; protein-protein interaction
Gan Y^49^	RegCS	III	IDD	Human	young vs adult healthy IVDs	chondroid ECM homeostasis;	IHC; immunofluorescence staining; flow cytometry; crystal violet staining; colony-forming unit-fibroblast activity; trilineage differentiation
***HomCs***							
Ji et al.^25^	HomCs	III	OA	Human	Different OA stages	act as the main controllers of the circadian clock in OA progression	IHC
Chou et al.^26^	HomCs	III	OA	Human	non-OA vs OA	in response to external stimuli; Regulation of gene expression	immunofuorescence staining; RT-qPCR; protein-protein interaction
Gan Y^49^	HomCs	III	IDD	Human	young vs adult healthy IVDs	delayed degeneration of IVDs	IHC; immunofluorescence staining; flow cytometry; crystal violet staining; colony-forming unit-fibroblast activity; trilineage differentiation
***HTC***							
Ji et al.^25^	HTC-A	III	OA	Human	Different OA stages	cartilage development and connective tissue development	IHC
	HTC-B					an extracellular matrix organization, ossification and mineralization	
Chou et al.^26^	HTC	III	OA	Human	non-OA vs OA	skeletal development; Multicellular organismal development	immunofuorescence staining; RT-qPCR; protein-protein interaction
	preHTC					ESkeletal system development;System development	
***Others***							
Ji et al.^25^	ECs	III	OA	Human	Different OA stages	energy supply	IHC
Chou et al.^26^	RepCs	III	OA	Human	non-OA vs OA	extracellular matrix signaling; collagen fibril organization	immunofuorescence staining; RT-qPCR; protein-protein interaction
	Pre-FCs					ECM organization and disassembly	
	FCs					Extracellular matrix organization; Movement of cell or subcellular component	
***Nucleus pulposus progenitor cells (NPPC)***							
Gao et al.^44^	*UTS2R*^*+*^ NPPC	III	IDD	Human/mice	Different grades of IDD	prevent IDD	IHC; immunofluorescence staining; Real-Time RT-PCR Analysis; flow cytometry; cell Proliferation Assay
Ling et al.^45^	*CD70*^*+*^*CD82*^*+*^NPPC	IV	IDD	Human	Different grades of IDD	proliferative ability; interactions with macrophages	NA
Gan Y et al.^49^	*ANGPT1*^*+*^NPPC	III	IVD	Human	young vs adult healthy IVDs	cell survival protection;	IHC; immunofluorescence staining; flow cytometry; crystal violet staining; colony-forming unit-fibroblast activity; trilineage differentiation
	*SOX9*^*+*^NPPC					cell proliferation	
	*PAX1*^*+*^NPPC					regulate NP homeostasis	
	*PDGFRA*^*+*^*PROCR*^*+*^NPPC					regulate NP homeostasis;cell differentiation lineages; the SMAD3 signaling pathway	
***Fibroblasts***							
Zhang et al.^71^	*THY1*^*+*^*CD34*^*−*^*HLA-DR*^*hi*^ fibroblast	III	RA or OA	Human	RA vs OA	mediator of RA pathogenesis	Histological assessment; multicolor immunofluorescent staining;flow cytometry
	*DKK3*^*+*^ sublining fibroblasts					prevention of cartilage degradation	
Stephenson et al.^76^	*CD55*^*+*^fibroblasts	III	RA	Human		protect the synovium from complex-mediated arthritis	immunofluorescence; flow cytometry
	*CD90*^*+*^ fibroblasts					organize the extracellular matrix	
Mizoguchi et al.^77^	*PDPN*^*+*^*CD34*^*–*^*THY1*^*+*^ fibroblasts	III	RA or OA	Human	RA vs OA	located around blood vessels in the synovium; joint destruction	Histological analysis; ELISA; Quantitative real-time PCR; osteoclastogenesis assay; Ki67 staining
	*CD34*^*–*^*THY1*^*+*^ fibroblasts					bone destruction in RA	
	*CD34*^*+*^ fibroblasts					monocyte recruitment in inflamed synovial tissue	
Croft et al.^78^	*FAPα*^*+*^*THY1*^*+*^ fibroblasts	III	OA	Mouse	Health vs OA	more severe and persistent inflammatory arthritis with minimal effects on bone and cartilage	CyToF; IHC; immunofluorescence staining; mass cytometry
	*FAPα*^*+*^*THY1*^*-*^ fibroblasts					mediated bone and cartilage damage with a limited effect on inflammation	
Zhou et al.^172^	*COL14A1*^*+*^matrix fibroblasts	III	Osteosarcoma	Human	Primary vs recurrent vs lung metastatic	exist in primary and recurrent lesions	IHC; immunofluorescence staining; immunofluorescence staining
	*MYL9*^*+*^*LUM*^*+*^fibroblasts					similar to myofibroblasts	
	*DES*^*+*^*COL14A1*^*-*^fibroblasts					detected in lung metastatic lesions	
***Macrophage***							
Chou et al.^26^	immune regulatory macrophage (IR-MΦ).	III	OA	Human	non-OA vs OA	clearance of cell remnants and degraded tissues and modulate immune responses of the synovium	immunofuorescence staining; RT-qPCR; protein-protein interaction
Culemann S et al.^73^	*CX3CR1*^*+*^tissue-resident macrophages	III	RA	Mice	Normal vs RA	formed a protective tight-junction-mediated barrier to physically isolate the joint and limit the inflammatory response	Flow cytometry and fluorescence-activated cell sorting; quantitative real-time PCR; HIC
Kuo et al.^74^	*HBEGF*^*+*^ inflammatory macrophages	III	RA	Human	OR vs RA	promote fibroblast invasiveness	qPCR; western blot
Aliverni et al.^75^	*MerTK*^*pos*^*CD206*^*pos*^ synovial tissue macrophage	III	RA	Human	Normal vs early/active RA	aid the restoration of synovial homeostasis	FACS; quantitative PCR; flow cytometry; immunofluorescence staining
Zhou et al.^172^	*FABP4*^*+*^ macrophages	III	Osteosarcoma	Human	Primary vs recurrent vs lung metastatic	contribute to pro-inflammation	IHC; immunofluorescence staining; immunofluorescence staining
Liu et al.^173^	*FABP5*^*+*^ macrophages	III	Osteosarcoma	Human	Normal vs Osteosarcoma	lipid metabolism	multiplex immunofluorescence staining
	*TXNIP*^*+*^ macrophages					similar to M2 polarization	
	*IFIT1*^*+*^macrophages					regulated regulatory T cells and participated in CD8 + T-cell exhaustion	
	*MKI67*^*+*^ macrophages					tissue-resident cells with proliferation ability	

*IDD* intervertebral disc degeneration, *IVD* intervertebral disc, *NPPCs* nucleus pulposus progenitor cells, *ProCs* proliferative chondrocytes, *preHTCs* prehypertrophic chondrocytes, *HTCs* hypertrophic chondrocytes, *ECs* effector chondrocytes, *RegCs* regulatory chondrocytes, *HomCs* homeostatic chondrocytes, *FCs* Fibrochondrocyte, *RepCs* reparative chondrocytes, *IHC* Immunohistochemical assays, *MRI* Magnetic resonance imaging, *PCR* polymerase chain reaction, *FACS* Fluorescence-Activated Cell Sorting, *CyToF* cytometry by time-of-flight

Nanus et al.^29^ identified seven distinct subgroups of synovial fibroblasts (Clusters 0-6) in patients with early- and end-stage OA. Cluster 0, with high expression of genes related to pain (*HSPA1A*, *DNAJB1*, *SLC39A8*, *HTRA3*, *ATF3*, *PTGIS*, and *BNIP3*), was more prevalent at the end stage of OA. At an early stage, Clusters 2-5, which expressed high levels of genes related to fibrosis, inflammation, and neuronal growth (*NFKBIA*, *CXCL2*, *GEM*, *VCAM1*, *LIF*, *IL-6*, and *INHBA*), were isolated from sites of pain in paIients with early-stage OA, whereas Clusters 1 and 6 were dominant in nonpainful regions. Cluster 1 was related to “role of *IL-17A* in RA,” regulation of “cell proliferation of fibroblasts,” “differentiation of neurons,” and “cellular viability of neurons.” Cluster 6 featured a reduction in neuronal cell viability and decreased “cell viability of CNS cells” and “cell survival.” Synovial fibroblasts in nonpainful synovial sites of patients with early OA were mainly observed in a single cluster. In contrast, fibroblasts from sites of pain in patients with early OA were more diverse and grouped into four distinct clusters. However, most fibroblasts were present in one large cluster in patients with end-stage OA, suggesting a shift during disease progression toward a simple OA-related fibroblast pathotype.^29^ Notably, the gene signatures of these end-stage OA fibroblasts were associated with eicosanoid and prostanoid signaling pathways that were known mediators of inflammation-related pain, which had provided a rationale for the use of nonsteroidal anti-inflammatory drugs as analgesics. Fibroblast-mediated protective mechanisms against inflammation and oxidative damage are gradually lost during OA progression.^29^

Immune cells, such as macrophages and T cells, associated effector cytokines, and cytokine receptors expressed by synoviocytes are involved in the development of OA.^30^ Macrophages, the most abundant type of immune cells, produce inflammatory cytokines, including TNF, IL-1β, IL-18, TGF-β1, and IL-8.^30^ Chou et al.^26^ found a novel IR-MΦ population with high expression of immunoregulatory genes that might play roles in clearing cell remnants and degraded tissues and in modulating immune responses in the synovium (Table 2). Sebastian et al.^31^ identified resident Lyve1^hi^Folr2^hi^ macrophages and Trem2^hi^Fcrls^+^ recruited macrophages that acted as chondroprotectors and aided in joint injury healing. T-cell-mediated inflammation has been considered less important than macrophage-mediated inflammation in OA pathogenesis. However, Huang et al. detected Th17 cells in the OA synovium, implying an important role for T cells in OA pathogenesis.^32^

Bian et al.^33^ performed scRNA-seq analysis of synovial joint progenitor cells in the developing murine knee joint from E12.5 to E15.5 and revealed that at E12.5, YFP, a nonfunctional fluorescent protein, was mainly expressed in the presumptive joint area, including part of the bone anlagen and the surrounding mesenchyme. At E13.5, YFP expression was sparse in the anlagen of the femur and tibia and was more centered in the interzone and the surrounding connective tissue. At E14.5, YFP was mainly present in the areas where articular cartilage, synovium, and surrounding soft tissue eventually developed. One day later, YFP was observed in the menisci, epiphyseal cartilage, and synovium. The lineage trajectories of Gdf5^+^ mesenchymal cells showed transcriptional heterogeneity at E12.5. One cluster tended to exhibit a more mesenchymal state, but another cluster tended to exhibit a more chondroprogenitor-like state.

**Fig. 2:**
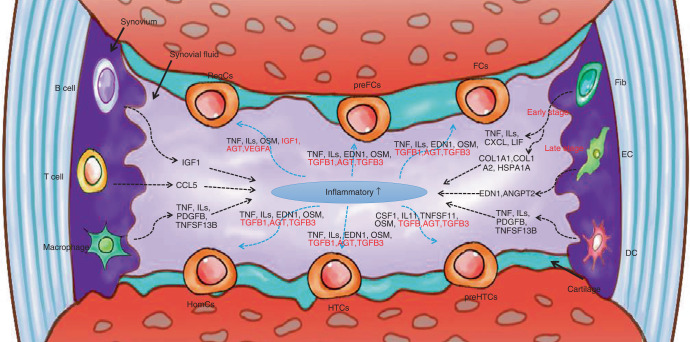
Overview of the crosstalk networks among the clusters in osteoarthritis. Fib fibroblast, EC endothelial cell, DC dendritic cell, RegCs regulatory chondrocytes, HomCs homeostatic chondrocytes, preHTCs prehypertrophic chondrocytes, HTCs hypertrophic chondrocytes, FCs fibrocartilage chondrocytes, preFCs prefibrocartilage chondrocytes

The advent of scRNA-seq has expanded our knowledge of how various cells, including subclusters of chondrocytes, fibroblasts, and immune cells, stimulate inflammation by secreting cytokines, proinflammatory factors, or chemokines, as shown in Fig. 2. However, the limited number of samples and lack of samples from healthy subjects may hinder further investigation of OA-related processes. Thus, researchers should share data and establish a public database. Although accumulating evidence describes the cellular heterogeneity of chondrocytes in patients with early- and end-stage OA, how macrophage or fibroblast subtypes exert pathological effects remains unknown, and their interactions with chondrocytes remain poorly understood. Encouragingly, scRNA-seq and other tools enable the precise identification of cellular diversity. In summary, the development of specific therapies for OA may require a two-phase approach that involves determining the functional roles of subpopulations and delineating the relevant molecular mechanisms.

### Application of scRNA-seq in lumbar disc herniation

**Fig. 3:**
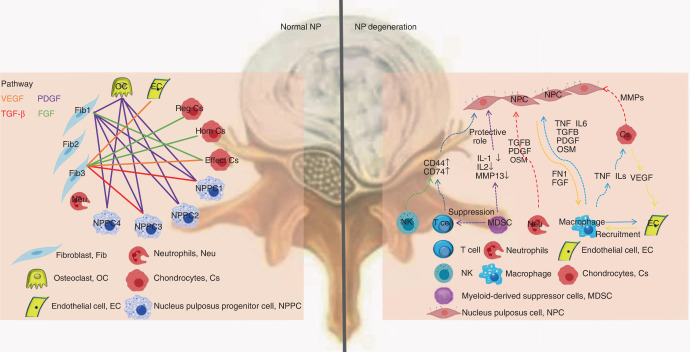
Overview of the crosstalk networks among the clusters in the normal normal nucleus pulposus (NP) and degenerative NP. Left: Cellular network regulating the homeostasis of the NP via VEGF, TGF-β, PDGF, and FGF signaling. Distinct colorful lines present the inferred VEGF, TGF- β, PDGF, and FGF signaling. Right: Diagram of the regulatory mechanisms of degenerative NP. Fib fibroblast, Neu neutrophils, OC Osteoclast, Cs Chondrocytes, EC endothelial cell, NPPC nucleus pulposus progenitor cell, NK nature killer, MDSC Myeloid-derived suppressor cells, NPC nucleus pulposus cell

LDH, affecting 1%-5% of the population per year,^34^ is commonly caused by intervertebral disc degeneration (IDD), which is involved in complex and multifactorial processes.^35,36^ IDD, characterized by dehydration of the central nucleus, annular disruption, and decreases in proteoglycan content, cellularity, and endplate density,^37^ is closely associated with the dysregulation of ECM homeostasis. The intervertebral disc (IVD) comprises the nucleus pulposus (NP) originating from the notochord, the annulus fibrosus (AF), and the cartilage endplate derived from the sclerotome.^38,39^ The cellular heterogeneity and molecular characteristics of IVD at various stages, which play an important role in improving treatment outcomes, remain poorly understood. Notably, scRNA-seq reveals new subpopulations in IVD as well as the functions of individual cellular subsets, which has improved our understanding of the pathological processes of IDD and provided new insights into therapeutic strategies (Fig. 3).

Furthermore, confirmation of the biomarkers of various IVD subpopulations has expanded our knowledge of the pathology of IDD. Cherif et al.^40^ used single-cell transcriptomics to identify specific biomarkers of NP cells and inner AF cells in individuals with and without disc degeneration. Wang et al.^41^ identified new marker genes of IVD cell types, including NP cells (*Krt7*, *Prrg4*, and *Akap12*), outer AF cells (*Myoc* and *Igfbp5*), and inner AF cells (*Bpifa2f*, *Mmp3*, and *IL11*), and proposed their functions in a healthy rat IVD model. In a healthy bovine IVD model, Panebianco et al.^42^ used scRNA-seq to identify numerous novel markers of NP cells (*CP*, *S100B*, *H2AC18*, *SNORC*, *CRELD2*, *PDIA4*, *DNAJC3*, *CHCHD7*, and *RCN2*), outer AF cells (*IGFBP6*, *CTSK*, *LGALS1*, and *CCN3*), and inner AF cells (*MGP*, *COMP*, *SPP1*, *GSN*, *SOD2*, *DCN*, *FN1*, *TIMP3*, *WDR73*, and *GAL*). The identification of biomarkers for various cell types improves IVD diagnosis and treatment.

Due to the complexity of IVD development, the cellular heterogeneity of IVD cells remains to be elucidated. Researchers have recently investigated cellular heterogeneity in IVD in distinct animal models and in humans by scRNA-seq. Panebianco et al.^42^ found that the NP3, NP5, outer AF2, and outer AF3 cell subsets might be potentially desirable subsets for exogenous cell therapies due to their ability to promote tissue repair by synthesizing ECM. The NP3, NP1, outer AF3, and outer AF2 cell subsets were found to contain IVD progenitor cells that might be targeted for endogenous repair. Calió M et al.^43^ combined scRNA-seq with RNA-seq to identify 27 NP structure/tissue-specific genes and 24 AF structure/tissue-specific genes from bovine calf tails. Additionally, a cluster of notochord-like cells acting as either regulators or progenitor cells was detected in the NP. In a mouse model, Gao et al.^44^ identified three NP cell subclusters, including transient NP cells that were mainly involved in the cytokine response, glycoprotein biosynthesis, and mesenchymal cell development, regulatory NP cells that were involved in major metabolic activities, and homeostatic NP cells with anti-inflammatory and anti-angiogenic functions. Ling et al.^45^ mainly identified novel subtypes of NP cells in human IVD. Homeostatic NP cells related to metabolism were mostly present in the early stage, whereas fibrocartilaginous NP cells associated with the cellular stress response were mainly present in the middle and late phases.^45^ Tu et al.^46^ identified effector NP cells that were enriched in metabolic process-related genes and positive regulation of ECM assembly, and homeostatic NP cells were related to cellular homeostasis. Additionally, regulatory NP cells were involved in the response to inflammation and endogenous stress, while adhesion-related NP cells were associated with cell migration and cell-matrix adhesion. CD90^+^ NP cells served as progenitor cells in degenerative NP tissues. These findings may greatly contribute to an improved understanding of tissue homeostasis and regeneration.^46^

The important role of nucleus pulposus progenitor cells (NPPCs) in the pathophysiological processes of IVD is well recognized,^47,48^ yet the subpopulations and their functions are unclear. Gan Y et al.^49^ identified four NPPC subclusters, including ANGPT1^+^ NPPCs related to cell survival and protection, SOX9^+^ NPPCs involved in cell proliferation, and PAX1^+^ NPPCs and PDGFRA^+^PROCR^+^ NPPCs that primarily regulated NP homeostasis. Notably, PDGFRA^+^PROCR^+^ NPPCs were found to have three differentiation lineages (osteogenic, chondrogenic, and adipogenic differentiation) due to the high expression of pluripotency-related genes (Table 2). Ling et al.^45^ identified CD70^+^CD82^+^ NPPCs with a high proliferative ability and observed their close interactions with macrophages. Gao et al.^44^ discovered a novel population of NPPCs (UTS2R^+^ NPPCs) that were located in the NP periphery and that might prevent IDD (Table 2). The IVD phenotype was determined by the fates of two NPPC subgroups (exhausted or altered during degeneration), implying the potential for novel treatment techniques targeting NPPCs^47,50,51^ (Table 2).

The proportion of chondrocytes in IVD is relatively low, but they exert important functions. In healthy human IVDs, a new set of regulatory chondrocytes with high growth factor and chondrogenic pathway regulators was found to play a regulatory role in chondroid ECM homeostasis^49^ (Table 2). Homeostatic chondrocytes have high expression levels of genes that regulate circadian rhythm, which is an important regulator of homeostasis.^49^ Additionally, effector chondrocytes play a crucial role in active metabolism by sustaining ECM biogenesis in IVD and have high expression levels of *PRG4*, which can reduce shear stress and inflammation.^49^ Zhang Y et al.^52^ also identified four novel subsets of chondrocytes (C1-C4) with different functions in human samples. C1 was associated with cytokine activity, and C2 was related to extracellular matrix structural (ECM) constituents. C3 and C4 were engaged in collagen binding, cell adhesion molecule binding, and integrin binding. Han et al.^53^ also identified four populations with different functions among patients. C1 and C3 were related to the inflammatory response. C2 expressed high levels of collagen type II and proteoglycan, which maintained the regular ECM structure. C4 had a phenotype comparable to that of fibroblasts and might play a role in ECM remodeling. The proportion of cartilage progenitor cells substantially decreased with the progression of IDD, whereas C3 cells were detected only in the severe IDD group.^53^

Immune cells, including T cells, natural killer (NK) cells, neutrophils, myeloid-derived suppressor cells, and macrophages, have been detected in degenerating NP but not in normal NP^46^ (Fig. 3). The proportion of M2 macrophages decreased with the progression of IDD followed by an increase in the proportion of M1 macrophages, indicating the crucial role of macrophage polarization in amplifying the inflammatory cascade during the process of IDD.^45^ A novel cell subtype, granulocytic myeloid-derived suppressor cells, was enriched at the middle stage and contributed to delaying NP cell degeneration by suppressing IL1-β and IL-2, which might be a potential treatment target for IDD^46^ (Fig. 3). SOX2^+^NGFR^+^ neurogenic cells have been identified in the NP as being related to neuronal growth. RUNX2 mainly contributes to postnatal IVD development by regulating the transition of notochord cells into chondrocyte-like cells.

Regarding the underlying mechanisms of IDD, heat shock protein genes are highly expressed in the process of disc degeneration, implying that stress-related mechanisms are associated with IVD.^46,47^ Zhang et al.^52^ reported that ferroptosis was distinctly activated in the mild IVD group in a rat model, which suggested that it might play a crucial role in the early stage of IDD, providing a new conceptual strategy for slowing IDD. Fernandes et al.^54^ mentioned that the inhibition of Wnt signaling was closely related to the onset of IDD. Furthermore, the TNF, MAPK, and Hippo pathways may promote disc degeneration.^46^

Cell‒cell interactions provide details regarding the underlying mechanisms in the development of IDD, and disruptions of these interactions may be a potential treatment for LDH. Han et al.^53^ explored the interactions between chondrocytes and macrophages as well as endothelial cells. Macrophages and endothelial cells can promote chondrocyte apoptosis and enhance the inflammatory response, whereas chondrocytes can stimulate the proliferation, migration, and differentiation of endothelial cells and accelerate macrophage recruitment, which may be key factors in the development of IDD.^53^ Ling et al.^45^ found strong cell interactions between M1 and M2 macrophages among different types of NP cells. Macrophage polarization from the M1 type to the M2 type regulates NPPC function through NPC via macrophage migration inhibitory factor and TGFβ signaling networks. According to the study conducted by Gan Y et al.,^49^ GF-related signaling pathway members, mainly including the FGF,^55,56^ TGF-β,^57,58^ BMP,^59,60^ VEGF, and PDGF^61^ families, are involved in the crosstalk network in the NP of human IVD (Fig. 3).

In summary, scRNA-seq technology has contributed to an overall understanding of the cellular heterogeneity and functions of individual cell subsets in IVDs, but research investigating IVDs remains at a preliminary stage. Some limitations and challenges regarding IDD research have been noted. First, due to the limited number of healthy human samples, the cellular heterogeneity and homeostasis in the NP, particularly the distinct differences between the outer and inner AF, remain poorly understood. An increase in data sharing and cooperation in this field is needed to overcome this limitation. Second, the predictive ability of scRNA-seq must be validated using traditional methodologies in future studies. Third, single-method studies have inherent limitations, so the combined application of scRNA-seq and other bioinformatics techniques will surely improve our understanding of NP and AF cell heterogeneity, interactions among cells, and key regulatory factors during IDD development and progression.

### Applications of scRNA-seq in rheumatoid arthritis

**Fig. 4:**
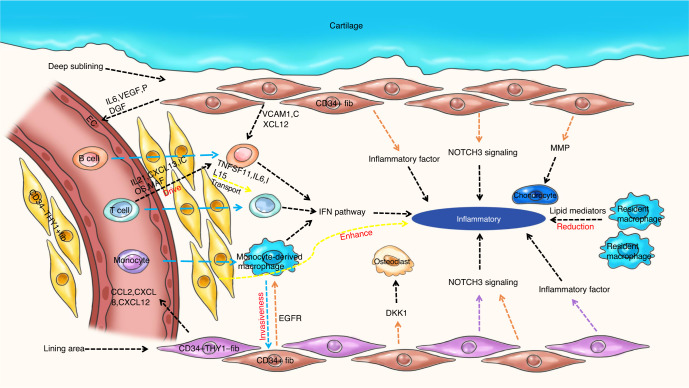
Overview of the crosstalk networks among the clusters in rheumatoid arthritis. Fib fibroblast, EC endothelial cell

RA, a common and chronic autoimmune inflammatory disease, affects 0.5% to 1% of the global population each year.^33^ RA is characterized by the destruction of synovial tissue by the host immune system. According to recent studies, the lining of the inflammatory synovium is thickened in individuals with RA, and immune cell infiltration is accompanied by the invasion of synovial fibroblasts, which are also related to the progression of RA.^62,63^ Increasing research has identified new subclusters, cytokines, and chemokines involved in the process of RA, as shown in Fig. 4. Here, we review the related research using scRNA-seq.

Currently, a few markers are used for the diagnosis and stratification of patients with RA in daily clinical practice. According to the presence or absence of anti-citrullinated peptide antibodies and/or rheumatoid factors, RA is divided into seropositive RA and seronegative RA.^64,65^ Recent articles have documented the roles of anti-carbamylated protein antibodies and anti-lysine acetylated antibodies in RA pathogenesis.^66–68^ Consequently, the broader phrase “anti-posttranslationally modified protein antibodies” may be used more frequently in future research and therapeutic situations. Bader L et al.^69^ applied mass cytometry to distinguish patients with RA from healthy donors and found that CD4, CD45RA, and CD11c identified CD4^+^ T-cell subsets and dendritic cells (DCs). Bader L et al.^69^ also identified p-p38, IκBα, p-cJun, p-NFκB, and CD86 as relevant and useful functional markers for the diagnosis and stratification of patients with RA. Another study^70^ discovered that most sequence variations exerted a greater effect on seropositive RA than on seronegative RA. A multiomics analysis revealed possible causative genes encoding proteins in the interferon-alpha/beta network and the JAK/STAT pathway. Zhang et al.^71^ observed newly identified inflammatory phenotypes aligned with RA treatments targeting the interferon pathway, JAK kinase inhibitors, and agents upregulating chemokines (*CXCL8, CXCL9*, and *CXCL13*), cytokines (*IFNG* and *IL15*), and surface receptors (*PDGFRB* and *SLAMF7*) in distinct immune and stromal cell populations, which provided new insights into the disease etiology and potential therapies. Similarly, according to the results of a receiver operating characteristic curve analysis, CCL2 and MMP13 were good predictive and diagnostic markers of RA.^72^

ScRNA-seq has been used to identify the heterogeneity of macrophages with different functions and biological characteristics. The relative proportions of synovial tissue macrophage subpopulations in patients with RA who are in remission might be a better biomarker than anti-citrullinated peptide antibodies for predicting persistent remission or disease flare, since anti-citrullinated peptide antibodies are rarely observed in clinical practice. Culemann S et al.^73^ showed a distinct population of epithelial-like CX3CR1^+^ tissue-resident macrophages forming a protective tight junction-mediated barrier to physically isolate the joint and limit the inflammatory response, and the numbers were maintained by a pool of locally proliferating CX3CR1^+^ mononuclear cells embedded in the synovial tissue, indicating an unexpected level of functional diversity among synovial macrophages (Table 2). Kuo et al.^74^ found that most medications used to treat patients with RA, such as nonsteroidal anti-inflammatory drug analgesics, targeted HBEGF^+^ inflammatory macrophages and were able to promote fibroblast invasiveness while still allowing them to transform into a classic proinflammatory M1-like phenotype, which presumably perpetuated inflammation. Studies investigating local macrophage morphologies and intercellular interactions have helped us better understand chronically inflamed human tissues and the effects of drugs (Table 2). In a study conducted by Aliverni et al.,^75^ therapeutic efficacy was enhanced by MerTK^pos^CD206^pos^ synovial tissue macrophage clusters induced by the activation of MerTK with agonists or by myeloid cell re-education induced by the activation of transcription factors driving a remission signature in synovial tissue macrophages (KLF2, KLF4, NR4A1, NR4A2, and ATF3), which could aid in the restoration of synovial homeostasis (Table 2).

Recently, scRNA-seq has revolutionized fibroblast research in RA. Stephenson et al.^76^ identified the following two subclusters: CD55^+^ fibroblasts and CD90^+^ fibroblasts (Table 2). The former was mainly located in the intimal lining layer and protected the synovium from immune complex-mediated arthritis, while the latter was located in the lower layer and facilitated ECM organization. Mizoguchi et al.^77^ found that a distinct subset of PDPN^+^CD34^–^THY1^+^ fibroblasts located around blood vessels in the synovium was expanded in individuals with RA and might be pathogenic (Table 2). Furthermore, the altered proportions of fibroblast subsets in patients with RA may disrupt homeostasis in the joint, leading to joint destruction. Zhang et al.^71^ identified two novel subclusters, including THY1^+^CD34^−^HLA-DR^hi^ fibroblasts with high *IL6* expression and DKK3^+^ sublining fibroblasts that were involved in preventing cartilage degradation (Table 2). Croft et al.^78^ found that the deletion of FAPα^+^ fibroblasts suppressed both inflammation and bone erosion. FAPα^+^THY1^*+*^ fibroblasts were mainly involved in more severe and persistent inflammatory arthritis with minimal effects on bone and cartilage, whereas FAPα^+^THY1^-^ fibroblasts selectively mediated bone and cartilage damage with a limited effect on inflammation. These differences have important implications for cell-based therapies aiming to modulate inflammation and tissue damage (Table 2). NOTCH3 signaling was revealed to play a key role in the differentiation and pathologic growth of CD90(THY1)^+^ sublining fibroblasts and led to the development of inflammatory arthritis, as described by Wei et al.^79^ The intermediate states of native human and mouse synovial tissue fibroblasts were partially replicated by NOTCH-activated fibroblasts in organoids. Importantly, the genetic deletion of *NOTCH3* or the blockade of NOTCH3 signaling with a monoclonal antibody reduced inflammation and protected joints from inflammatory arthritis. The aforementioned findings established a molecular foundation for therapeutically targeting sublining fibroblasts in individuals with RA by modulating NOTCH3 signaling.^79^

Other immune cells, such as T cells, NK cells, eosinophils, monocytes, and B cells, also play crucial roles in the development of RA. Kelkka et al.^80^ detected a specific TCR signature in CD8^+^ cytotoxic T cells from patients with aggressive anti-citrullinated peptide antibody-RA and high expression of the bone destruction-associated cytokine TNFSF14. Rao et al.^81^ found that PD-1^hi^CXCR5^-^CD4^+^ T cells expressing high levels of *CXCL13* and *IL-21* mainly contributed to the recruitment of B cells and PD-1^hi^CXCR5^+^ T follicular helper cells. An understanding of tissue-localized T and B-cell interactions might yield a new RA treatment approach.^81^ As shown by Fonseka et al.^82^, CD27^-^HLA^-^DR^+^ effector memory cells with Th1^-^ and cytotoxicity-associated features were enriched at the primary sites of inflammation and were related to the efficacy of treatment. Han et al.^83^ observed a connection between active IFN signaling pathways and disease promotion by CD56^bright^ and CD56^dim^ NK cells in subjects with RA. Activation of the type I IFN signaling pathway might generate the inflammatory signaling amplifier CH25H. The IFN signaling pathway is stimulated in individuals with RA, potentially impacting antibody class switching and autoantibody synthesis in RA plasma cells. Gene regulatory networks were shown to be important for the mechanism of transcriptional regulation, suggesting that further studies investigating IFN signaling pathways in patients with RA are warranted. According to Andreev et al.^84^, a novel subset of regulatory eosinophils that resided in the joints expressed high levels of proresolving *5-LOX* and *12/15-LOX*, and these cells were able to resolve chronic arthritis via the ILC2-IL-5 axis at the remission stage in RA. The accumulation of regulatory eosinophils in the joints also enhanced joint structure preservation, indicating that regulatory eosinophils may facilitate tissue regeneration. The regeneration-promoting effects of these cells were mediated by the release of resolvins, the modulation of macrophage polarization, and the activation of synovial tissue regeneration. Zhang et al.^71^ found that IL1B^+^ proinflammatory monocytes were potential key mediators of RA pathogenesis. Meednu et al.^85^ identified unique B cells that were enriched in the RA synovium and expressed a high level of *NR4A*, which may be considered a biomarker of chronic autoantigen stimulation in the synovium and a novel therapeutic target. The HLA-DR15 haplotype was identified by Wu et al.^86^ as a risk factor for the development of active disease in patients with anti-citrullinated peptide antibodies^+^ RA. Inflammatory myeloid cells, such as M1 macrophages and MMP3-secreting DC subsets, play crucial roles in synovial pathogenesis in anti-citrullinated peptide antibody^-^ RA synovial tissue macrophages, whereas lymphoid cells (B cells and T cells) play a prominent role in anti-citrullinated peptide antibody^+^ RA synovial tissue macrophages.^86^ Wu et al.^86^ discovered that the immunological pathogenesis differed between patients with anti-citrullinated peptide antibody^-^ and anti-citrullinated peptide antibody^+^ RA, which contributed to an improved understanding of the histology, heterogeneity, progression, and treatment of RA.

Additionally, several studies have revealed the differences between OA and RA using scRNA-seq. Liao et al.^72^ found that fibroblasts (Cluster 0), enriched in the OA group, were related to protein processing in the endoplasmic reticulum, glycolysis/gluconeogenesis, and proteoglycans in cancer. However, Cluster 3, which was involved in focal adhesion, ECM receptor interactions, and phagosomes, was enriched in the RA group.^86^ Liao et al.^72^ also discovered significantly fewer B cells in the RA group, while the numbers of CD8^+^ T cells and neutrophils were substantially higher. Cai et al.^87^ reported similar potential regulatory effects of the Wnt signaling pathway, the TGF signaling pathway, the FcRI signaling pathway, and the ERBB signaling pathway on synovial fibroblasts from individuals with RA and OA, indicating potentially overlapping pathogenic mechanisms in these two diseases and potentially revealing new therapeutic targets to attenuate disease progression.

In summary, scRNA-seq not only reveals the heterogeneity, marker genes, and signaling pathways of chondrocytes, synovial cells, and immune cells but also enables identification of the lineage and/or developmental relationships among these cells in individuals with RA. Some impressive research results have been obtained, yet scRNA-seq technology still has infinite value in future research on RA. Unidentified populations of cells, including synovial cells, osteoblasts, endothelial cells, and mesenchymal cells, might be hidden and crucial factors contributing to the development of RA. In addition, changes in cellular subsets, including phenotypes and marker genes of articular cells at different developmental stages, should be further identified. More in-depth studies of new subpopulations and the differentiation and dynamic evolution of cells are needed. The combination of scRNA-seq technology and multiomics might be effective for clarifying the intercellular networks and pathological mechanisms that contribute to the development of RA. Traditional biological methods, including flow cytometry, lineage tracing, cell function experiments, and knockout experiments in animals, should be used to rigorously verify some key cell populations and important molecular events. The development of a scRNA-seq platform might lead to additional breakthroughs in RA treatment.

### Applications of scRNA-seq in fractures

**Fig. 5:**
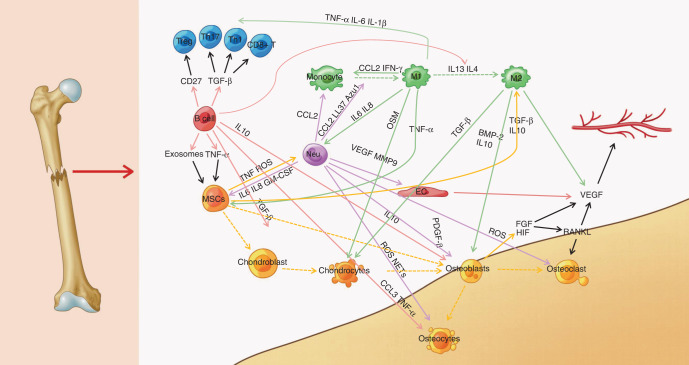
Overview of the crosstalk networks among the clusters in fracture. EC endothelial cell, M macrophage, MSC mesenchymal stem cell, Treg regulatory T cell, Th helper T cell

Fractures, which impact approximately 3.21 out of every 1 000 individuals each year, are a major contributor to mortality and disability worldwide.^4,88^ Bone nonunion is estimated to occur in 1.9% of all cases of adult fractures, and the risks increase with age in patients older than 60 years.^89–91^ The functions and quantities of osteoblasts and osteoclasts directly affect fracture healing.^91,92^ An inflammatory reaction precedes the creation of a fibrous callus, the deposition of cartilage, and the generation of bone tissue, which is remodeled to restore the original shape and function of the injured bone. Endochondral ossification occurs when cartilage is replaced by bone, and skeletal stem/progenitor cells, whose generation is triggered by bone damage, preferentially develop into chondrocytes in the center of the callus.^93,94^ A better understanding of the functions of different stromal cell subtypes, including MSCs, committed osteoblast lineage cells, mature osteocytes, chondrocytes, vascular cells, and bone-degrading osteoclasts (Fig. 5), in bone formation and bone healing would inform potential strategies for treating fractures.

Based on accumulating evidence, bone mesenchymal stromal cells represent a heterogeneous cell group composed of different cell populations with distinct molecular and functional properties.^95–97^ However, researchers have not determined whether bone mesenchymal stromal cell subsets are derived from several different cell populations or from a single stem/progenitor cell population. An understanding of the regulatory factors controlling the differentiation and lineage specification of bone mesenchymal stromal cells is important. Sivaraj et al.^98^ applied scRNA-seq to investigate the function, lineage differentiation, and cell fate transition of bone mesenchymal stromal cells and found that diaphysis mesenchymal stromal cells with limited differentiation potential were related to the sinusoidal vasculature in the bone microenvironment, while metaphysis mesenchymal stromal cells had multilineage (osteogenic, adipogenic, and chondrogenic) differentiation potential. Furthermore, the fate of bone mesenchymal stromal cells was controlled by PDGFRb signaling and the transcription factor Jun-B, which were involved in stromal cell proliferation and differentiation during bone formation.

Previous studies have revealed the contribution of skeletal muscle to bone repair.^99–102^ Skeletal muscle is associated with impaired healing, and muscle flap coverage may clinically improve bone healing. However, the underlying mechanisms remain poorly understood.^103–106^ Julien et al.^107^ reported that Prx1-derived skeletal muscle mesenchymal progenitors in cartilage and bone directly contributed to bone repair. Within 3 days after a fracture, skeletal muscle mesenchymal progenitors with high expression of the chondrogenesis regulator gene *Sox9* were predominantly in a fibrogenic state and then shifted toward a chondrogenic phenotype until 5 days post-fracture. However, in the event of polytrauma (injury to both bone and skeletal muscle), skeletal muscle mesenchymal progenitors exhibited altered fibrogenesis and chondrogenesis, which directly impaired bone healing. At later stages of repair, damaged skeletal muscle adjacent to the bone fracture was responsible for fibrous tissue accumulation within the fracture callus, interfering with fracture consolidation. Thus, antifibrotic drugs may be further developed for therapeutic use, laying a solid foundation for the enhancement of bone repair and the prevention of pathological fibrosis.

A proper bone microenvironment formed by immune cells plays a critical role in fracture healing during the early inflammatory stage.^108^ Immune cells have been reported to disrupt fracture repair by decreasing the immunogenicity of mesenchymal stromal cells.^109^ However, the effect of immune cells on fracture healing has not yet been fully clarified. Zhang et al.^110^ applied scRNA-seq to observe immune cells in mouse fracture models and detected a significant difference in B cells but no marked differences in other immune cells. B cells were the least abundant population at the callus formation stage and the most abundant population at the callus healing stage because B cells played a crucial role in the differentiation of osteoblasts and osteoclasts, indicating that B cells were important contributors to fracture healing. Furthermore, exosomes secreted from B cells decreased osteogenic activity by inhibiting osteoblast differentiation and promoting osteoclast formation, so exosomes might be an alternative nanomedicine option in the treatment of fractures. Further elucidation of the relationship between bone formation and B-cell development may provide new potential therapeutic targets. Avin et al.^111^ compared the proportions of immune cells and the difference in the expression of their markers by scRNA-seq and found higher proportions of monocytes and CD14^+^ DCs, as well as lower proportions of T cells, myelocytes, and promyelocytes in the nonunion bone group. Additionally, the related gene expression changes provided insights from an osteoimmunological perspective. These findings enable a solid understanding of the process of bone nonunion.

In summary, although immune cells and stromal cells have been suggested to play important roles in fracture healing, the variations in their numbers or types at the three stages of fracture healing remain poorly understood. Future studies should use scRNA-seq combined with other techniques to assess the key mechanisms and the dynamic variations in immune cells and stromal cells at all stages of bone healing, which might increase the efficacy of treatments promoting fracture healing. Additionally, further research on the interactions between stromal cells and immune cells during fracture healing might be helpful in providing new therapeutic targets.

### Applications of scRNA-seq in tendon injuries

Tendon injuries are one of the most common traumas in clinical practice, affecting approximately 1 in 1 000 individuals.^5^ Long-term and excessive loading of tendons may lead to tendon ruptures and degenerative tendinopathies because the low numbers of resident tendon fibroblasts limit the capacity for repair.^112^ Tendons, consisting of type I collagen, elastin, proteoglycans, various matrices, tendon fibroblasts, and tenocytes,^112,113^ are dense connective tissues that allow forces to be efficiently transmitted.^114,115^ Studies investigating the cellular heterogeneity and detailed molecular mechanisms that control the dynamic differentiation processes might improve our understanding of the complicated biological functions, which might facilitate the creation of new remedies.^116^ To the best of our knowledge, most studies have focused on morphological or mechanical changes. However, the various cell types in mature tendons, the developmental trajectory of tendon differentiation, and their gene expression profiles remain largely unknown. Recently, numerous articles have investigated cellular heterogeneity by scRNA-seq. Here, we review the latest research investigating tendons through the application of scRNA-seq.

By performing a trajectory inference analysis, De Micheli et al.^117^ identified three novel subclusters of fibroblasts, including tendon fibroblast Clusters 1 and 2 and junctional fibroblasts, which were mainly involved in the production of tendon ECM components. These researchers also found that three subpopulations expressed high levels of *Spp1*, *Dpt*, and *Smoc2*, which were involved in ECM binding. De Micheli et al.^117^ also found that pericytes might be progenitor cells of adult fibroblasts in the tendon. Furthermore, analyses of cell‒cell interactions showed that TGF signaling and CTGF signaling played important roles in tendon development and homeostasis.^117^

Still^118^ identified four subclusters of tendon progenitor cells with distinct functions in response to a mechanical stimulus. Pro-inflammatory tendon progenitor cells were associated with the inflammation-mediated pathology of tendinitis. SLC40A1^+^ tendon progenitor cells were involved in tendon development and general wound repair. Mechanically responsive tendon progenitor cells were mainly enriched in the healthy samples. NES^+^ clonogenic tendon progenitor cells were mainly abundant in the disease samples, enabling a better understanding of tendon progenitor cells and the tissue.

Due to poor vascularization, tendons are rarely self-renewing, and a high reinjury rate after reconstructive surgery using autologous tendons. Hence, an overall understanding of tendon differentiation and molecular pathways is needed to develop strategies for restoring tendon function. Fan et al.^119^ identified the regulatory role of a nerve growth factor-secreting Cd9^+^Cd271^+^ tendon stem/progenitor cluster in tendon maturation and the NGF/SHP2 pathway. Nakajima T et al.^120^ used scRNA-seq to analyze the developmental trajectory of induced pluripotent stem cell-derived tenocytes and found that cell grafting contributed to efficient motor function recovery after Achilles tendon damage in rats, implying that human pluripotent stem cell-derived tenocytes had therapeutic potential in the context of tendon injury. At 19 days after injury, a staggering 91.6% of cultured cells were positive for *Scx*, a pivotal gene responsible for tenocyte development, indicating that Nakajima’s model recapitulated the normal stepwise progression and narrowing of fate decisions during paraxial mesoderm development (inducing the differentiation of induced pluripotent stem cells toward tenocytes).^120^ Yoshimoto et al.^121^ also performed scRNA-seq analysis to investigate the tenogenic differentiation trajectory and revealed a progressive trajectory in which cells transition from Scx^+^/Tnmd to Scx^+^/Tnmd^+^, Scx/Tnmd^+^, and finally Scx/Tnmd^+^ phenotypes. Activating retinoic acid signaling could decrease TGF-dependent early tenogenic development, as evidenced by a lower number of Scx^+^ cells, whereas suppressing retinoic acid signaling increased both tenogenic and fibrochondrogenic differentiation. These findings provide valuable information for expanding our understanding of tendon biology.^121^ By activating the TGF and Hedgehog pathways, Kaji et al.^122^ built directed differentiation models by creating tendon and fibrocartilage cells from mouse embryonic stem cells with an induction efficiency of 90% and performed scRNA-seq to clarify the mechanisms. Retinoic acid signaling is considered a vital regulator of the cell fate shift between TGF-induced tenocyte and fibrocartilage cell development. As shown in a study conducted by Kult et al.^123^, cells in tendon-to-bone attachments had dual fates. They exhibited transcriptomic features similar to both chondrocytes and tenocytes and were regulated by common regulatory elements and Krüppel-like transcription factors, which acted as regulators of cell attachment. Furthermore, the inhibition of Krüppel-like factor-2 and Krüppel-like factor-4 expression in the developing limb mesenchyme suppressed differentiation.

Garcia-Melchor et al.^124^ explored the direct interaction between tenocytes and T cells and observed T cells in normal tendons with a greater proportion in tendinopathy. After coming into direct contact with tenocytes after tendon injuries and full activation, T cells exert an effect on tenocytes. Furthermore, tenocytes upregulated genes implicated in inflammation, T-cell recruitment, and migration, creating a vicious cycle that may contribute to the emergence of a chronic inflammatory response. These findings may reveal a novel translational strategy for the management of tendon disorders.^124^ De Micheli et al.^117^ found that immune cell-fibroblast or pericyte-fibroblast interactions mainly involved TGF signaling and CTGF signaling.

In summary, ongoing studies are exploring the tendon phenotype, cellular heterogeneity within tendons, trajectory of tendon differentiation, and molecular mechanisms of tendon diseases, helping us obtain a better understanding of the tendon healing process and the process of tendon degeneration. The use of scRNA-seq technology to investigate how mechanical stress affects distinct cellular populations in healthy and diseased tendons would almost certainly have ramifications for the development of tendon therapies. Regarding the tendon phenotype, various markers, such as *TNC*, *SXC*, and *COL1A1*, have been used to define tendons, but these genes are not specific to tendons.^125,126^ The identification of unique markers of the tendon through a combination of techniques is urgently needed. Further confirmation of key regulatory genes, proteins, and pathways involved in tendon/fibrocartilage differentiation and cellular interactions between tenocytes and immune cells might lead to the development of potential treatments for tendon diseases. Gain- or loss-of-function assays should also be conducted to determine how transcription factors affect tendon and fibrocartilage specification. Further mechanistic research using an in vivo tendon damage model might provide insights into the importance of the adaptive immune response in tendon pathogenesis.

### Applications of scRNA-seq in spinal cord injury

SCI, which affects 1.3‰ of the general population, is a severe injury of the central nervous system that leads to a loss of motor and sensory function.^6,127^ Basic research has revealed three key mechanisms involved in the pathophysiology of SCI. The first barrier is the penumbra of the hypertrophic lesion, which arises after central nervous system injury and contains one of three inhibitory cellular components that obstruct axon regeneration at peripheral nervous system-to-central nervous system graft contacts and inside the lesion parenchyma. The fibrotic scar is the second barrier to regeneration and is located near the lesion penumbra but inside the glial scar.^128,129^ The third is the lesion epicenter, which mainly includes systemically derived inflammatory cells.^130^ Cell transplantation therapy has recently been shown to be effective in SCI animal models.^131,132^ The application of scRNA-seq in SCI research has provided novel insights into the cellular and molecular heterogeneity as well as structural alterations in the central nervous system following trauma.

Microglia, which are resident macrophages, play a crucial role in the rapid response to injury.^133^ Li et al.^130^ identified five clusters of microglia (MG0-MG4). MG0 was associated with microglial homeostasis in the intact spinal cord, MG1 was associated with typical microglial activation, and MG2 was the intermediate state between MG1 and MG0. Notably, genes related to the ECM, wound healing, phagocytosis, angiogenesis, fibronectin binding, and negative regulation of immune responses were only expressed in MG3, implying that the MG3 cluster may have the ability to promote scar-free healing. Furthermore, research has revealed that neonatal microglia secreted fibronectin and binding proteins to form ECM connections that ligated the severed ends of the spinal cord, expressed several extracellular and intracellular peptidase inhibitors that improved healing and axon regrowth in microglia, and expressed other molecules involved in resolving inflammation.^130^ In contrast to adult microglia, neonatal microglia remain permanently active to undergo a rapid and transient conversion to a homeostatic state, which is critical for scar-free wound healing.^130^ Wahane et al.^134^ discovered four different types of microglial cells, including an immunity-focused MG1 population, a reactive MG2 population, an immediately responsive MG3 population, and a proliferative MG4 population. After SCI, a continuous cellular trajectory began with the activation of the MG3 cluster in the immediate response, progressed to the immunity-focused MG1 cluster and reactive MG2 cluster, and then to the proliferative MG4 cluster, which was mainly influenced by HDAC3 activity. Wang et al.^135^ discovered four subpopulations of microglial cells: interferon-responsive microglia; homeostatic microglia; injury-associated microglia related to interferon production, cytokine production, lipid metabolism, gliogenesis, glial cell migration, fibroblast proliferation, and antigen presentation; and proliferation-associated microglia involved in oxidative stress and proliferation. Hakim et al.^136^ discovered a disease-linked Myc^+^Glmp^+^Nfe2l2^+^Skil^+^ microglial subcluster that evolved from permanently reprogrammed baseline microglia via an active cell state. These cells emerged days after a severe injury and remained in the human spinal cord at the lesion site for years. This subcluster appeared to play a role in healing and resembled those observed in the context of neural degeneration or demyelination, and a specific microglial cell type was identified throughout development. Microglial cells have been demonstrated to play a crucial role in coordinating the damage response using scRNA-seq. In future investigations, other molecular components involved in restoring homeostatic microglia should be identified and their functions should be assessed.

Similar to microglial cells, macrophages are important for spinal cord repair. Milich et al.^137^ found two subtypes of macrophages, including chemotaxis-inducing macrophages associated with the chemotaxis of other leukocytes and inflammatory macrophages linked to glial and macrophage activation and the inflammatory response. Wahane et al.^134^ identified four subpopulations of macrophages (Mac1-4). Mac1 was involved in iron and collagen binding, angiogenesis, lipid metabolism, and phagocytosis, while Mac2 was related to M1 macrophage activation. Mac3 was associated with prostaglandin synthesis and receptors for advanced glycation end product activation. Mac4 was engaged in ECM interaction and Hippo-Merlin signaling and exhibited a persistent reparative function due to the high expression of phagocytic genes, anti-inflammatory hallmark genes, ECM genes, and trophic factors. Research regarding immune cells, particularly macrophages, that are involved in SCI is still in its initial stages. Limiting the development of proinflammatory macrophages without interfering with the beneficial effects of macrophages on wound healing will be challenging.

In the central nervous system, neural stem cells, located in the ependyma in the central canal region, have a low proliferation rate for self-renewal under normal conditions, but they have great potential for proliferation and generation after SCI.^138^ Shu et al.^139^ discovered a novel group of nestin^-^GFP^+^ neural stem cells originating from outside the central canal. In the intact spinal cord, these cells were homogenous, but they were activated (with proliferation and differentiation abilities) 5 days after SCI, suggesting that they might serve as a source for regenerative treatment for SCI in the future. In a study involving single-cell transcriptome analysis conducted by Stenudd et al.^140^, highly differentiated ependymal A cells and a relatively rare subpopulation of spinal cord ependymal cells were defined as having stem cell properties with the ability to undergo self-renewal after SCI, which provided novel insights into regenerative therapeutic interventions for spinal cord repair.

An increasing number of studies have investigated the dynamic changes in cellular clusters after SCI, which improves our understanding of the overall development of SCI. The findings reported by Li et al.^130^ showed that 28.2% of microglial cells divided immediately after injury, and this percentage decreased to 15.1% and 9.5% at three and five days after injury, respectively. One day after injury, a clear gap between the two stumps of the cut spinal cord was observed. Microglial cells and fibronectin^+^ cells appeared in the gap two and three days after injury, respectively.^130^ Three days after injury, when fibronectin^+^ bridges formed between the two stumps, activated microglia were observed inside the lesion, but there were no GFAP^+^ astrocytes or Col1a1^+^ fibroblasts. Seven days after injury, the fibronectin signal was no longer present, and serotonergic axons had grown into and covered the injury.^130^ According to a study conducted by Hakim et al.^136^, baseline microglia underwent a rapid shift immediately after SCI (0-2 h), shifting to an equilibrium state between 6 and 36 h, and then gradually achieved a steady state (ninety days after injury). Milich et al.^137^ found that neutrophils, monocytes, and chemotaxis-inducing macrophages were the most prevalent populations one day after injury, but inflammatory and chemotaxis-inducing macrophages were the most numerous cells seven days after injury.^137^ Although astrocytes and oligodendrocyte progenitor cells initially exhibited comparable general responses to injury, they developed distinct functional identities and many functions formerly associated with reactive astrocytes that were attributed to oligodendrocyte progenitor cells seven days after injury.^137^ Three days after injury, Wang et al.^135^ observed a higher proportion of injury-associated cDCs that were involved in the inflammatory response and oxidative phosphorylation, migratory DCs that were engaged in antigen presentation, acute injury-associated neutrophils that were related to the induction of inflammation, phagocytosis, glial cell activation, fibroblast proliferation, and axon regeneration, and proliferation-associated microglia that were related to oxidative stress and proliferation. In contrast, cDC2s, mature neutrophils that proliferated and played a role in angiogenesis, γδ T cells that almost became activated Th17 cells, and injury-associated microglia with functions similar to those of injury-associated neutrophils were predominant fourteen days after injury. Additionally, Hakim et al.^136^ described dynamic alterations in the expression of various genes at three, seven, and fourteen days after injury. Three days after injury, the cell cycle and interferon signaling were the main enriched pathways, whereas migration and ion channel activity were activated seven days after injury, and ECM reorganization was observed fourteen days after injury.^136^ Cancer metastasis, mitotic kinase, and interferon signaling pathways were enriched three days after injury, whereas fourteen days after injury, pathways related to the activation of LXR/RXR, deactivation of synaptic long-term depression, opioids, Gβγ, and calcium signaling were enriched.^136^ Replicating all types of cellular interactions in SCI models and their alterations over time are critical. These findings might improve our understanding of the development of SCI and provide novel insights into the treatment of SCI.

Here, we reviewed scRNA-seq-related discoveries regarding complicated structures with an emphasis on the cellular heterogeneity of microglia, macrophages, neural stem cells, and variations of these cell types, as well as gene expression patterns over time after injury. Therapies that limit further secondary damage, increase the number of stem-like cells, and resolve uncontrolled inflammatory processes are urgently needed. These areas are hot topics and potential targets for the future. The application of multiomics technology might deepen our understanding of the heterogeneous composition of the scar and its evolution over time to accelerate research into axon regeneration and functional recovery. Ideally, the optimal treatment will concurrently address both the intrinsic problem (the decrease in axon outgrowth machinery components) and the extrinsic obstacle (the increase in axon inhibitory proteins) to facilitate targeted rehabilitation.

### Applications of scRNA-seq in heterotopic ossification

HO, which affects 20% of patients with forearm fractures,^7^ results from a diverse and complicated pathological process involving soft tissues, such as muscle, peri-articulations, and ligaments.^141–144^ The unique cellular source is one of the most notable differences between pathological HO and normal osteogenesis.^145^ Evidence suggests that the local activities of macrophages, monocytes, and other cells at prospective HO sites result in significant increases in inflammatory cytokine levels.^146^ These factors cause the mobilization and recruitment of certain stem cell populations^147^ as well as the local activation of pro-skeletogenic signaling processes,^148^ leading to the activation of chondrogenesis and osteogenesis and the accumulation of ectopic bone tissue. However, the cells of origin related to HO pathogenesis are unknown. The application of scRNA-seq in HO research has improved our understanding of the cellular origins, pathophysiology, and underlying processes of the disease.

Chen et al.^149^ first identified two subclusters of classified pathological cells in an ectopic ossification model. HO1 cells were similar to chondrocytes, while HO2 cells were similar to tenocytes with high expression of *Sox9* and *Scx*, which were involved in the migration of connective tissue between the tendon and bone.^150,151^ Pseudotemporal analysis suggested that HO2 cells were the precursors of HO1 cells. ATAC-seq and scRNA-seq analyses revealed that Xbp1, which was a key effector protein of endoplasmic reticulum stress and was positively related to the activity of the Notch, Wnt, Hh, and TGF-β signaling pathways, was enriched in HO2 cells but enriched to a lesser degree in HO1 cells. These findings may be attributed to the crosstalk of these pathways in ectopic ossified tissues.^149^

Activin A, a member of the TGF superfamily, regulates various essential physiological processes involving immunological responses, wound healing, inflammation, and fibrosis.^152,153^ According to Hwang et al.^154^, the inhibition of activin A is not a therapeutic strategy for treating posttraumatic HO. Hwang et al.^154^ assessed the expression of *activin A* in response to injury and found that *activin A* was expressed in different types of cells following injury in subjects with two primary forms of HO, posttraumatic HO and fibrodysplasia ossificans progressiva.^154^ Moreover, an anti-activin A agent did not block the development of posttraumatic HO, whereas antibodies that neutralized ACVR1 or ALK3-Fc, which blocked osteogenic bone morphogenetic protein expression, were beneficial for patients with HO.^154^ Nevertheless, Mundy et al.^155^ discovered that blocking endogenous activin A could reduce HO aggravation and bone accumulation by decreasing the recruitment of Sox9^+^ skeletal progenitors expressing *activin A (Inhba)* to sites of HO. Furthermore, activin A enhanced the chondrogenic differentiation of progenitor cells through SMAD2/3 signaling, and the inclusion of activin An in HO-inducing implants enhanced HO development in vivo, indicating that this was an effective therapeutic strategy for forms of this pathology in patients. Barruet et al.^156^ found that activating activin receptor type-1 induced the differentiation of pluripotent stem cell-derived muscle stem/progenitor cells or human muscle stem cells, which might lead to HO by altering the local tissue environment.

Accumulating studies have revealed the functions of small molecules in HO. Hsu et al.^157^ discovered that the expression of *Wisp1*, a member of the CCN family, was positively associated with bone and cartilage repair in mouse and human HO. *Wisp1* expression was negatively correlated with trauma-induced HO, and pathways associated with enhanced cartilaginous differentiation have been implicated in transgenic *Wisp1*-knockout animals. These results provide more information on the roles of CCN family signaling in HO and may contribute to the development of targeted therapies.^157^ According to previous findings, the expression of another crucial factor, *TAK1*, is triggered by BMP signaling and downstream noncanonical (SMAD-independent) BMP signaling.^158–160^ In a study conducted by Strong et al.^161^, an inhibitor of *TAK1* (NG-25) limited ectopic bone formation following blast-associated lower limb trauma in models in vivo and in vitro, implying the critical role of TAK1 signaling in chondrogenic differentiation and HO. Lin et al.^162^ used *Mkx*-knockout mice as an HO model to investigate the potential mechanisms and found that BIBF1120, an inhibitor of angiogenesis, significantly decreased bone formation and vascularity in *Mkx*^−/−^ mice, uncovering a pathogenic mechanism of HO and a novel treatment option. According to a study conducted by Qin et al.^163^, the inhibition of axonal ingrowth suppressed angiogenesis and HO development by modulating neuron-to-vascular signaling, and the researchers identified a regulatory role for nerve-to-vessel crosstalk in the pathology of HO. Sorkin et al.^164^ revealed an important role for TGF-β1^*+*^ monocytes/macrophages at the initial stage of HO through single-cell transcriptome analysis and found that they inhibited CD47 in an HO mouse model.

Currently, the clinical diagnosis of HO is commonly based on X-ray or computed tomography. However, these two methods do not identify mature HO until 6 weeks after trauma, at which point prospective therapies are ineffective.^165–167^ Edwards et al.^168^ distinguished the location of soft tissue endochondral ossification at early stages through high-frequency spectral ultrasound imaging, such that effective interventions could be applied when and where they were needed after injury. Furthermore, the chondrogenic to osteogenic transition occurred in mesenchymal progenitor cells during HO and was correlated with gene expression, as revealed by an analysis using high-frequency spectral ultrasound imaging for the detection of HO anlagen. ScRNA-seq analysis revealed that mesenchymal progenitor cells were responsible for HO, and early tissue changes in matched chondrogenic and osteogenic gene expression were detected using spectral ultrasound imaging.

In summary, although numerous articles have focused on the roles of cytokines, transcription factors, and inhibitors in HO, further functional experiments are still needed to explore specific functional mechanisms. In addition, the crosstalk and regulatory relationships among signaling pathways, including the Wnt, Notch, BMP, Hh, TGF-β, SMAD2/3, and TAK1 signaling pathways, must be further validated to confirm the pathway activation patterns at the single-cell level. Spectral ultrasound imaging can detect the location of soft tissue endochondral ossification in the early stages, laying the foundation for effective early intervention. Novel techniques must be developed to improve the accuracy of distinguishing early HO sites from local tissue. Furthermore, we should identify the dynamic variations in cell subsets, gene expression, and underlying mechanisms in HO cells, chondroblasts, and myocytes or immune cells at all stages by scRNA-seq combined with spatial transcriptome analysis.

### Applications of scRNA-seq in osteosarcoma

**Fig. 6:**
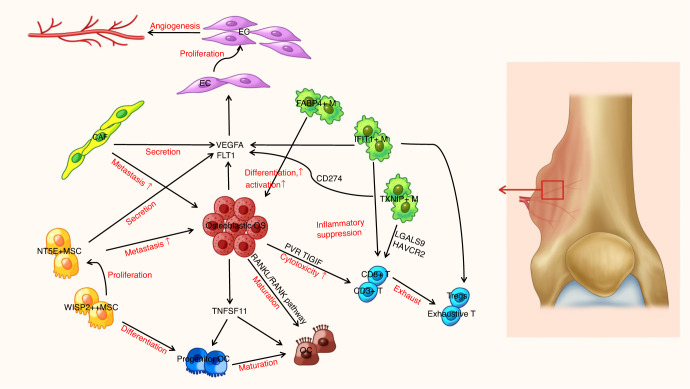
Overview of the crosstalk networks among the clusters in osteosarcoma. OS osteosarcoma, CAF tumour associated fibroblast, EC endothelial cell, OC osteoclasts, M macrophage, MSC mesenchymal stem cell, Treg regulatory T cell

OS, with a high relapse rate and a low survival rate, is a highly aggressive and malignant bone tumor.^169,170^ In the 1990s, gene therapy became a popular research topic, and related studies have provided novel insights into the treatment of OS. The successful treatment of OS requires an accurate and effective diagnosis, preoperative chemotherapy, surgical resection, postoperative chemotherapy, and life-long monitoring. Ongoing studies have revealed the interactions among diverse cell types, including osteoblasts, fibroblasts, and immune cells, and their functions in the development of OS,^171^ as shown in Fig. 6. However, the cellular heterogeneity and molecular mechanism remain poorly understood. ScRNA-seq analyses enable us to better understand the underlying mechanisms of the development and progression of OS.

Osteoblastic OS cells are a major cell type contributing to conventional OS in the clinic. Zhou et al.^172^ identified six subclusters of osteoblastic OS cells. Clusters 1 and 2 corresponded to the original cluster of proliferating osteoblastic cells. Cluster 3 was associated with angiogenesis and the IFN-α and IFN-γ signaling pathways. Cluster 4 was enriched in the MYC and oxidative phosphorylation signaling pathways. Cluster 5 was engaged in the TGF-β, P53, KRAS, and hypoxia signaling pathways. Cluster 6 was linked to myogenesis, inflammatory responses, and allograft rejection signaling pathways. Liu et al.^173^ identified five osteoblastic OS subpopulations (C1-C5) with distinct functions in patients with naive osteosarcoma. C1 was related to inflammation, while C2 was associated with the cell cycle and cell proliferation. C3 was involved in carbohydrate transmembrane transporter activity and glucose catabolic processes. C4 was enriched in the response to ECM processes and protein import into the peroxisome matrix. C5 participated in replacement ossification, bone trabecula morphogenesis, and bone trabecula formation. Furthermore, the modulation of osteolysis by osteoblastic cells via nuclear factor kappa-B ligand‒receptor activator was discovered. Osteoblastic OS cells also regulated angiogenesis via vascular endothelial growth factor-A. In particular, a high level of C1/5 osteoblastic OS cells was linked to a poor prognosis, suggesting that they might have a higher clinical value than other cell types in terms of possible therapeutic uses.^173^

Osteoclasts are considered to play vital roles in osteolysis and tumor growth.^174^ Osteoclasts are the only cells in the human body that are known to undergo resorption by bone. The findings reported by Liu et al.^173^ identified four subclusters, including progenitor osteoclasts, mature osteoclasts, hypofunctional osteoclasts, and nonfunctional osteoclasts, and revealed that osteoclast differentiation and function were regulated by osteoblastic OS cells through the TNFSF11-TNFRSF11A interaction.^173^ Zhou et al.^172^ detected three osteoclast subclusters. Progenitor osteoclasts were related to the stimulation of osteogenesis, while immature osteoclasts and mature osteoclasts played an important role in advanced OS. However, there was a lower proportion of mature osteoclasts in the chondroblastic, lung metastatic, and recurrent lesions than in the primary OS lesions, implying that the osteoclast status might depend on the strength of the chemotactic signal during osteogenesis in OS lesions.

Cancer-associated fibroblasts, which can stimulate tumor progression, growth, and metastasis, are regarded as an important component of the tumor microenvironment.^175^ Zhou et al.^172^ identified three subclusters of cancer-associated fibroblasts, including COL14A1^+^ matrix fibroblasts, DES^+^COL14A1^-^ fibroblasts, and MYL9^+^LUM^+^ fibroblasts. DES^+^COL14A1^-^ fibroblasts might be smooth muscle-like cells, whereas MYL9^+^LUM^+^ fibroblasts were similar to myofibroblasts. COL14A1^+^ matrix fibroblasts and MYL9^+^LUM^+^ fibroblasts were mainly observed in primary and recurrent lesions, while DES^+^COL14A1^-^ fibroblasts were mostly detected in lung metastatic lesions (Table 2). Liu et al.^173^ also identified three subclusters (C1-C3) of cancer-associated fibroblasts. C1 was associated with vascular remodeling, vascular development, and vascular diameter size. C2 was involved in osteoblast proliferation, development, and ossification. C3 was associated with the cell cycle and cell proliferation.

Immune cells, such as macrophages and T cells, also greatly contribute to the treatment of OS. Tissue-resident macrophages may be related to OS progression because FABP4^+^ macrophages, which may contribute to inducing inflammation, were detected in lung metastatic OS lesions^172^ (Table 2). M1 tumor-associated macrophages displayed increased activity of signaling pathways stimulated by IFN-α, IFN-γ, IL2/STAT5, IL6/JAK/STAT3, and inflammatory responses, indicating that these macrophages were generated from a proinflammatory microenvironment in OS lesions induced by IFN-α and IFN-γ. The TGF-β and Hedgehog signaling pathways were more active in M1 tumor-associated macrophages, suggesting their potential involvement in M2 polarization.^172^ A study conducted by Liu et al.^173^ identified five subtypes of macrophages. FABP5^+^ macrophages were related to lipid metabolism, while TXNIP^+^ macrophages were similar to M2 macrophages. FIT1^+^ macrophages that were associated with M1 polarization regulated regulatory T cells and participated in CD8^+^ T-cell exhaustion, illustrating the possible effectiveness of immunotherapy targeting CD8^+^ T cells and macrophages. MKI67^+^ macrophages might be tissue-resident cells with the ability to proliferate (Table 2). T cells are crucial components of cancer immunotherapy,^176^ but their heterogeneity in terms of cell types, gene expression patterns, and functional characteristics has a significant effect on the response to T-cell-based immunotherapy. CD8^-^CD4^-^ T, CD8^+^ T, CD4^+^ T, Treg, proliferating T, and NKT cells were discovered by Zhou et al.^172^ Recurrent and metastatic OS lesions contained a lower percentage of CD4^+^ and CD8^+^ T cells than primary lesions, which might indicate the ineffectiveness of T-cell-based immunotherapy.^172^ Liu et al.^173^ found that IFIT1^+^ macrophages interacted with exhausted T cells and Tregs via chemokine ligand receptors. These cells may be applicable in immunotherapy for OS.

Anti-TIGIT treatments have recently attracted increased interest as checkpoint therapies because they can modulate the activities of CD8^+^ T, Treg, and NK cells.^177–180^ *TIGIT* was highly expressed in Treg, CD8^+^ T, CD4^+^ T, and NKT cells from patients with OS, as revealed by scRNA-seq, suggesting that TIGIT inhibitor therapies may benefit patients with OS.^173^ Zhou et al.^173^ also utilized a TIGIT blocking antibody to confirm the cytotoxicity of CD3^+^ T lymphocytes toward OS cells, providing preliminary evidence that TIGIT inhibition might be a viable treatment strategy for OS in the future. Qin et al.^181^ observed that downregulation of *GZMB* was related to prolonged survival in patients with OS,^182^ and upregulation of *ITGA1* was positively associated with cancer status^183–186^ in the ATG16L1^+^ CD8 T-cell population. These findings implied that *ATG16L1* might be a prognostic biomarker and a therapeutic target in OS.

In summary, substantial advances in OS therapies have been made over the past decade. Despite various new medications and treatment methods, an improved understanding of the mechanisms regarding treatment success and failure is needed. Therefore, in-depth analysis of the molecular profiles and the mechanisms underlying drug resistance in various phases of OS must be performed using scRNA-seq along with other tools. These findings might provide beneficial information for the development of diagnostic strategies and therapeutic regimens.

### Other emerging technologies

Single-cell techniques enable researchers to analyze cellular subpopulations and molecular events at the single-cell level instead of the total cell population, which has substantially expanded our knowledge of orthopedic disorders. These techniques not only reveal cellular subpopulations that regulate homeostasis but also elucidate the subpopulations that drive disease. In this section, we only briefly review some emerging technologies for single-cell analysis, including transposase-accessible chromatin using sequencing (ATAC-seq), spatial transcriptome, and multiomics assays, because these methods have been described in detail in previous studies.^187–189^

ATAC-seq defines open and closed chromatin across the genome in specific cell types.^190,191^ Single-cell ATAC-seq (scATAC-seq) is a single-cell resolution method for studying the key regulatory regions in the genome and marking the epigenome of cell subtypes, which improves the understanding of cell type-specific regulatory regions. Recently, scATAC-seq has been used in some orthopedic diseases, such as SCI.^192^ However, important methodological and computational obstacles limit its applications in orthopedic diseases, such as chondrocytes.^188^ Great effort should be made to discover new approaches for advanced evaluations of regulatory activity based on scATAC-seq data to more accurately reconstruct gene regulatory networks, as the understanding of such networks is crucial for assessing the effect of genetic regulatory variants on disease risk at the individual cell level.

The absence of spatial information during single-cell isolation is a limitation of single-cell transcriptomics. The advent of spatial transcriptomics, which provides unique positional barcodes to visualize RNA distribution based on RNA sequencing data from tissue sections, overcame this obstacle.^193^ However, to the best of our knowledge, spatial transcriptomics is mainly applied in brain and tumor research but is rarely used in orthopedic research, potentially due to its high cost and impracticality. From our perspective, spatial transcriptomics could be applied for IDD, OA, RA, and meniscus injury research in future studies, which may provide new insights into the diagnosis, treatment, and prevention of orthopedic diseases.

Omics, mainly genomics, transcriptomics, and proteomics, provide data on orthopedic pathogenesis at the one-dimensional level.^194^ The advent of single-cell omics has provided unprecedented insights into the development of diseases, allowing us to better understand the features of genetic information transmission and gene expression at a higher throughput. For example, single-cell whole-genome sequencing is a useful technology for identifying single-cell nucleotide polymorphisms. Single-cell multiomics has broad development prospects. First, it can be used to identify cell subtypes from various cell populations. Second, cell lineage tracing can provide novel insights into the development of diseases.

In summary, emerging single-cell analysis methods have great potential for use in orthopedic research because they can provide nonoverlapping information about diseases. We may be able to better identify different populations in heterogeneous tissues, discover transcriptomic changes and their origins, and quantify and evaluate multiple samples simultaneously to provide new perspectives for the personalized treatment of orthopedic disorders.

### Summary and perspectives

Herein, we reviewed the recent advancements in orthopedic research by using scRNA-seq. This method has revealed new markers and cell subtypes, functions of novel cell types, potential mechanisms related to disease, cell-fate transition, and cell‒cell interactions in some common orthopedic-related disorders, including OA, LDH, RA, fractures, tendon injuries, SCI, HO, and OS, expanding our understanding of the development of these disorders. Although substantial progress has been made in orthopedic studies through the use of scRNA-seq, a comprehensive understanding of the cellular heterogeneity and molecular mechanisms underlying the development and homeostasis of orthopedic diseases remains in the preliminary stage. For example, to our knowledge, no report has focused on osteoporosis, which is a very common and important orthopedic disease influencing numerous people, particularly in the elderly population.

ScRNA-seq has been very popular in recent years, yet there remain some limitations regarding its use in orthopedic research. The first limitation is that it is difficult to prepare single-cell suspensions from bone, cartilage, and muscle tissue without changing the transcriptional profile. It is difficult to extract single cells from cartilage and especially from bone due to the hardness of these tissues. Both bone and cartilage tissue are digested by collagenase after dissection, but a longer digestion step is needed for bone samples. Additionally, to isolate sufficient amounts of RNA with sufficient quality, scRNA-seq of bone or cartilage tissue requires a larger sample than is the case with other tissues because bone and cartilage contain a smaller number of cells per unit volume than other tissues. Thus, it is difficult to apply this technique in small animal studies. Although multiple displacement amplification is a better method for genomic DNA amplification, especially in clinical samples with low DNA quantities, it has some drawbacks, such as amplification bias and unbalanced genome coverage.^195^ In terms of muscle samples, single-nucleus RNA sequencing enables us to resolve the obstacle of ‘big cells’, such as myocytes. Second, batch effects and systematic mistakes are common limitations. Sample handling, the use of various batches of chemicals, and the use of several biological specimens may all cause batch effects. Different computational tools, such as ComBat, could be used to correct these issues, but the need for such corrective tools should ideally be avoided by establishing proper experimental designs, analyzing multiple biological replicates, or pooling cells across experimental conditions and samples with subsequent demultiplexing using a cell tagging strategy.^195–198^ Third, the high cost of this technology results in a limited number of samples being analyzed, which reduces the significance of studies or perhaps even hinders their broader application in orthopedic research. Multicenter collaborations and an intergovernmental organization that combines orthopedic research and infrastructural resources are needed to overcome these challenges. Fourth, scRNA-seq is widely used by various groups, and these single-center data have obvious limitations. However, the sequencing data reported in some papers in the field of orthopedics remain undisclosed or unshared, which impedes further advancements. Researchers in this field should share data and cooperate to guide further studies.

Similarly, several challenges persist in orthopedic research, which hampers the transmission of valuable information to support clinical decision-making. One challenge is the obvious bias and deviations in the results of sequencing, cell clustering, and cell population identification studies. The potential reasons include individual differences in clinical samples, methods for choosing samples and isolating cell samples, the level of bioinformatics analysis, and the researchers’ understanding of cell markers. To address this problem, researchers should use appropriate standards or potential methods for reaching a widely recognized expert consensus in future orthopedics research. Another challenge is the successful and meaningful clinical interpretation of complex high-dimensional single-cell multiomics data, which requires the interdisciplinary collaboration of bioinformaticians, computational scientists, biologists, and clinicians. User-friendly data analysis databases and standard file formats can be created in the future, which would alleviate the difficulties of streamlining analysis and data sharing.

ScRNA-seq has facilitated great advances in orthopedic research and provided new potential treatment approaches for common diseases in the clinic. From our perspective, the following steps will drive further advancements in the application of scRNA-seq in orthopedic research. First, attention should be given to the dynamic variations in the pathophysiology of orthopedic disorders and the microenvironment of tissues to identify the regulatory subpopulations or disease-specific subpopulations, as well as the interactions among various subclusters at the single-cell level to identify key signaling pathways, particularly those between tissue cells and immune cells. Second, the combination of scRNA-seq with other emerging technologies will not only address the limitations of each technique but also yield nonoverlapping information on the identity of cells as well as enable more detailed and accurate cell classifications, greatly expanding our understanding of orthopedic diseases and providing potential treatments for them. We should be mindful in considering that much more rigorous verification must be conducted using traditional biological technologies, such as flow cytometry, lineage tracing, cell function research, and knockout animal experiments. Third, the acquisition of scRNA-seq data from single centers inevitably limits the scope and depth of orthopedic studies. We hope that researchers worldwide will share data and cooperate to drive the advancement of orthopedics research. As stated before, we believe that the continued application of scRNA-seq technologies will yield more breakthroughs in orthopedic research.
